# Cannabidiol promotes apoptosis via regulation of XIAP/Smac in gastric cancer

**DOI:** 10.1038/s41419-019-2001-7

**Published:** 2019-11-07

**Authors:** Soyeon Jeong, Min Jee Jo, Hye Kyeong Yun, Dae Yeong Kim, Bo Ram Kim, Jung Lim Kim, Seong Hye Park, Yoo Jin Na, Yoon A Jeong, Bu Gyeom Kim, Hassan Ashktorab, Duane T. Smoot, Jun Young Heo, Jeongsu Han, Sun Il Lee, Han Do Kim, Dae Hyun Kim, Sang Cheul Oh, Dae-Hee Lee

**Affiliations:** 1 Department of Oncology, Korea University Guro Hospital, Korea University College of Medicine, Seoul, 08308 Republic of Korea; 2 Graduate School of Medicine, College of Medicine, Korea University, Seoul, 08308 Republic of Korea; 3 Department of Medicine, Howard University, Washington, District of Columbia 20060 USA; 4 Department of Medicine, Meharry Medical Center, Nashville, Tennessee 37208 USA; 5 Department of Medical Science, School of Medicine, Chung-nam National University, 266, Munhwa-ro, Jung-gu, Daejeon, 35015 Republic of Korea; 6 Department of Surgery, Korea University Guro Hospital, Korea University College of Medicine, Seoul, Republic of Korea; 7 Kaiyon Bio Tech Co., Ltd, 226 Gamasan-Ro, Guro-gu, Seoul, 08308 Republic of Korea; 8 Department of Marine Food Science and Technology, Gangneung-Wonju National University, Gangwon, 210-702 Korea

**Keywords:** Gastric cancer, Apoptosis

## Abstract

According to recent studies, Cannabidiol (CBD), one of the main components of *Cannabis sativa*, has anticancer effects in several cancers. However, the exact mechanism of CBD action is not currently understood. Here, CBD promoted cell death in gastric cancer. We suggest that CBD induced apoptosis by suppressing X-linked inhibitor apoptosis (XIAP), a member of the IAP protein family. CBD reduced XIAP protein levels while increasing ubiquitination of XIAP. The expression of Smac, a known inhibitor of XIAP, was found to be elevated during CBD treatment. Moreover, CBD treatment increased the interaction between XIAP and Smac by increasing Smac release from mitochondria to the cytosol. CBD has also been shown to affect mitochondrial dysfunction. Taken together, these results suggest that CBD may have potential as a new therapeutic target in gastric cancer.

## Introduction

Cannabidiol (CBD) is one of the *Cannabis sativa* extracts that does not contain psychoactive components and is considered more useful than tetrahydrocannabinol, a psychotropic active cannabinoid, in clinical applications^1,2^. CBD is known to have antitumor activity against Noxa activation, inhibition of mTOR/cyclin D1, and G-protein-coupled receptors/mitogen-activated protein kinase pathway in various cancers such as pancreatic^3,4^, glioblastoma^1^, colorectal^5^, and breast cancer^6^. It also has beneficial effects on brain function, metabolism, and pain reduction^7–9^. However, there have been few reports on the anticancer mechanism by CBD in gastric cancer.

Apoptosis is a form of programmed cell death that plays an important role in many intracellular organs, such as growth, development, and homeostasis^10^. During apoptosis, the inhibitor of apoptosis (IAP) family of proteins including X-linked IAP (XIAP), c-IAP1, and c-IAP2 directly inhibit caspases and regulate cell death, among which XIAP exhibits the most potent anti-apoptotic effect^11,12^. XIAP has three baculoviral IAP repeat (BIR) domains (BIR1, BIR2, and BIR3) and a RING (really interesting new gene) finger domain at the C-terminal. The BIR2 domain is important for inhibiting caspase-3 (Cas3) and Cas7, and the BIR3 domain is essential for inhibiting Cas9. The Ring domain induces proteasomal degradation by promoting ubiquitination of other proteins and self-ubiquitination as well^13,14^.

The main negative regulator of XIAP is the second mitochondria-derived activator of caspase (Smac). Smac is normally present in the mitochondria and when apoptosis occurs it is released into the cytosol, and the signal peptide of the N-terminal region is removed and becomes an active form^15^. Activated Smac then competitively blocks the caspase-binding sites of XIAP and induces the caspase cascade, resulting in further apoptosis^16^.

Usually, XIAP has higher expression in cancer tissues than in normal tissues, whereas Smac has lower expression in cancer tissues than in normal tissues, both of which are associated with poor prognosis and survival rate of patients. Moreover, it has been reported that XIAP and Smac are negatively correlated in various cancers such as renal cell carcinoma and non-small cell lung cancer^16–18^. However, the underlying mechanism is still not thoroughly understood.

In the present study, we investigated the modes and molecular mechanisms of programmed cell death by CBD on gastric cancer cells. We have demonstrated for the first time that CBD induces apoptotic cell death by XIAP/Smac. Our results show that CBD induces mitochondrial dysfunction and regulates Smac/XIAP leading to apoptosis, suggesting that Smac/XIAP regulation using CBD can be potentially utilized for the treatment of gastric cancer.

## Results

### CBD enhances apoptotic cell death on gastric cancer

**Fig. 1:**
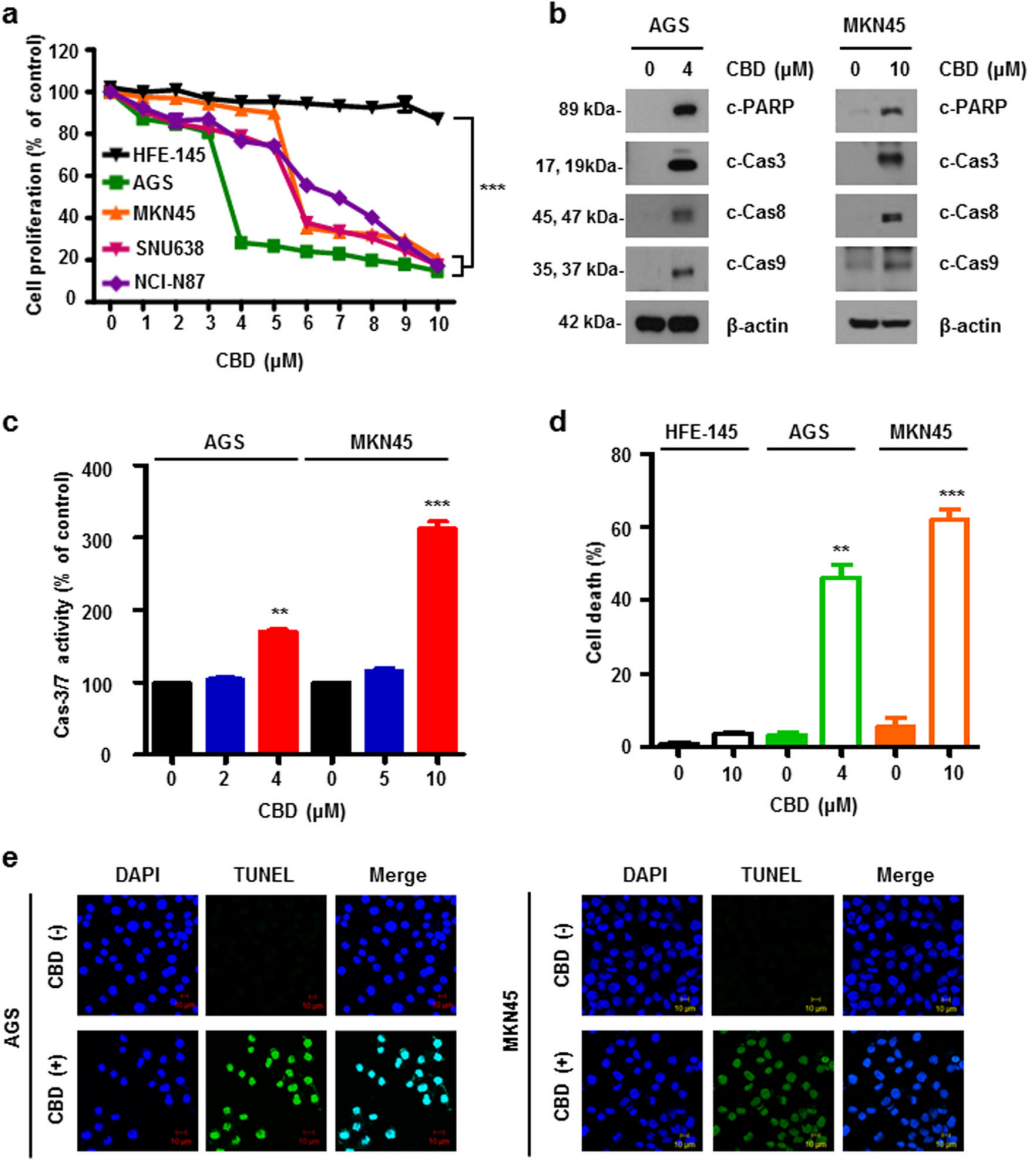
CBD increases cell death on gastric cancer. **a** HFE-145, AGS, MKN45, SNU638, and NCI-N87 cells were treated with 0 to 10 μM CBD for 24 h. Cell proliferation was examined by WST assay. ***P < 0.001. **b** CBD was treated in AGS and MKN45 cells for 24 h. Expression of c-PARP, Cas3, 8, and 9 were evaluated by western blotting. **c** Caspase a**c**tivity was measured by staining with IncuCyte Cas3/7 Reagent for 30 min in AGS (left) and MKN45 (right) treated with CBD for 24 h. **P < 0.01 and ***P < 0.001. **d** Cell death was determined by flow cytometry using Annexin V/PI staining. **P < 0.01 and ***P < 0.001. **e** Representative images of AGS (left) and MKN45 (right) cells stained with TUNEL. Green fluorescence indicated apoptotic signals

To investigate cell proliferation with CBD treatment, we performed a WST-1 assay following CBD treatment in various gastric cancer cells including AGS, MKN45, SUN638, and NCI-N87, and normal gastric HFE-145 cells (Fig. 1a). We found that cell proliferation decreased following CBD treatment, but it had no effect in gastric normal cells. Western blotting analysis was performed to determine the level of apoptosis after CBD treatment in AGS and MKN45 cells. As a result, the expression of cleaved- poly (ADP-ribose) polymerase (c-PARP) and Cas3, Cas8, and Cas9, both markers of apoptosis, were increased (Fig. 1b). To specifically confirm the increase of caspases, we measured Cas3/7 activity using a Caspase-Glo 3/7 Assay kit. As shown in Fig. 1c, caspase activities are elevated in CBD treatment condition. Moreover, to assess apoptosis, we measured Annexin V/propidium iodide (PI) staining using flow cytometry. CBD caused apoptosis in both AGS and MKN45 cells but not in HFE-145 cells (Fig. 1d). To verify this, a TdT-mediated dUTP nick-end labeling (TUNEL) assay was performed to stain apoptotic cells. As a result, the expression of TUNEL-positive cells was increased during CBD treatment in AGS and MKN45 cells (Fig. 1e). Based on these results, it can be concluded that CBD causes apoptosis in gastric cancer cells.

### CBD induces apoptosis by downregulating XIAP

**Fig. 2:**
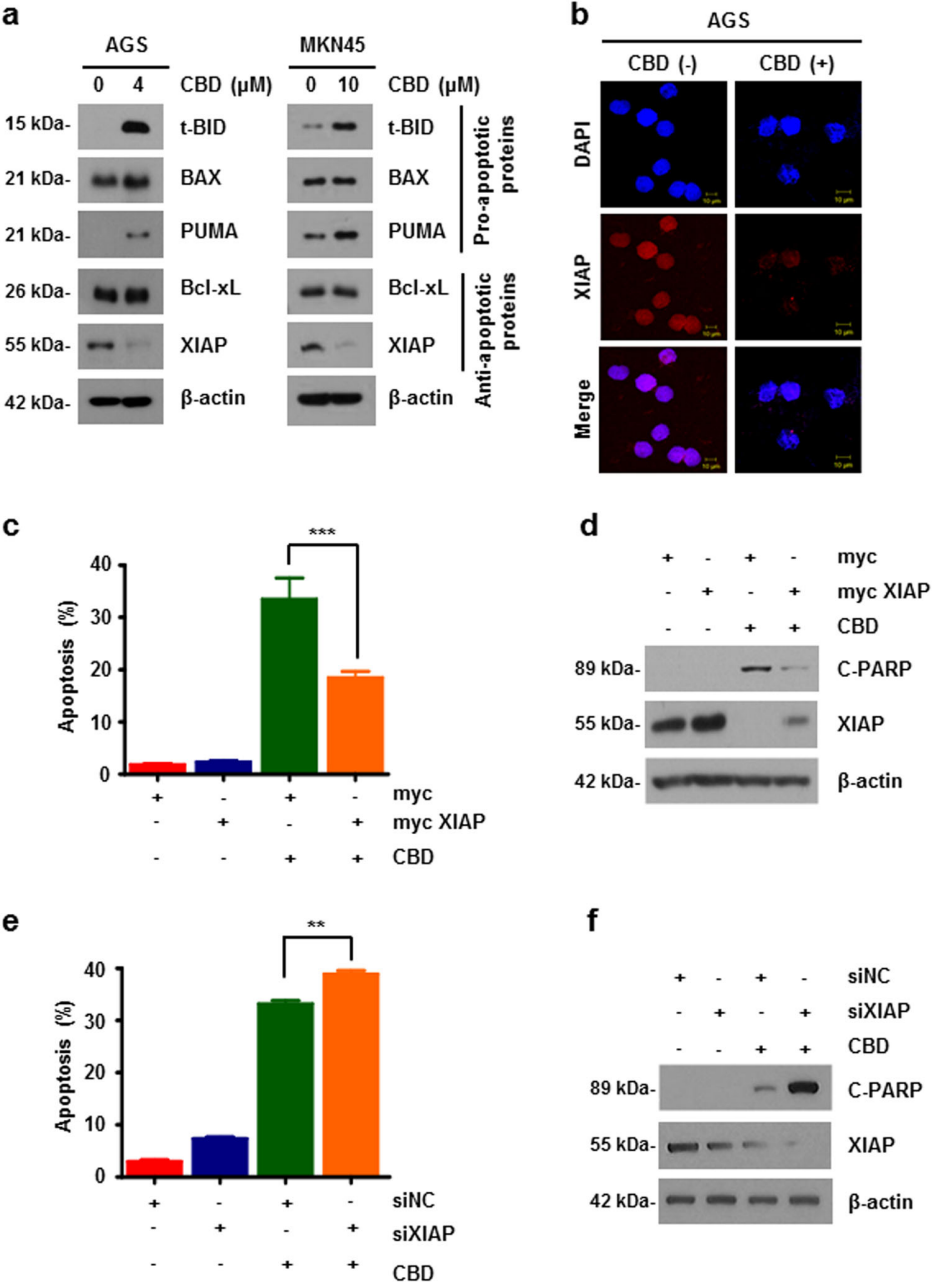
CBD suppresses expression of XIAP. **a** AGS (left) and MKN45 (right) cells treated with CBD for 24 h. Cells were collected for western blotting with the indicated antibodies. **b** AGS cells were treated with 4 μM of CBD for 24 h. Cells were immunostained with anti-XIAP (Red). Images were obtained using a confocal microscope. **c**, **d** AGS cells were transfected with myc-XIAP plasmid and treated with 4 μM CBD for 24 h. Apoptosis of cells was determined by flow cytometry (**c**) and immunoblotting (**d**). ***P < 0.001. **e**, **f** XIAP was knocked down using siRNA, treated with 4 μM CBD for 24 h, and subjected to flow cytometry (**e**) and west**e**rn blotting (**f**)

Apoptotic proteins were screened to determine which of them were involved in CBD-induced apoptosis. The expression of XIAP was remarkably reduced as measured with western blotting (Fig. 2a). To confirm the expression level of XIAP, an immunofluorescence assay was performed in AGS cells treated with CBD. As shown in Fig. 2b shown, XIAP expression decreased in the CBD treatment condition. Conversely, an experiment was conducted to determine whether apoptosis was affected or not in XIAP overexpression conditions. As a result, it was confirmed using western blotting and flow cytometry that when XIAP was overexpressed, apoptosis was rescued (Fig. 2c, d). Furthermore, knockdown of XIAP by small interfering RNA (siRNA) exacerbated CBD-induced apoptosis (Fig. 2e, f). These results revealed that downregulation of XIAP in CBD-treated gastric cancer cells induces apoptosis.

### CBD reduces XIAP through the ubiquitin-proteasome system

**Fig. 3:**
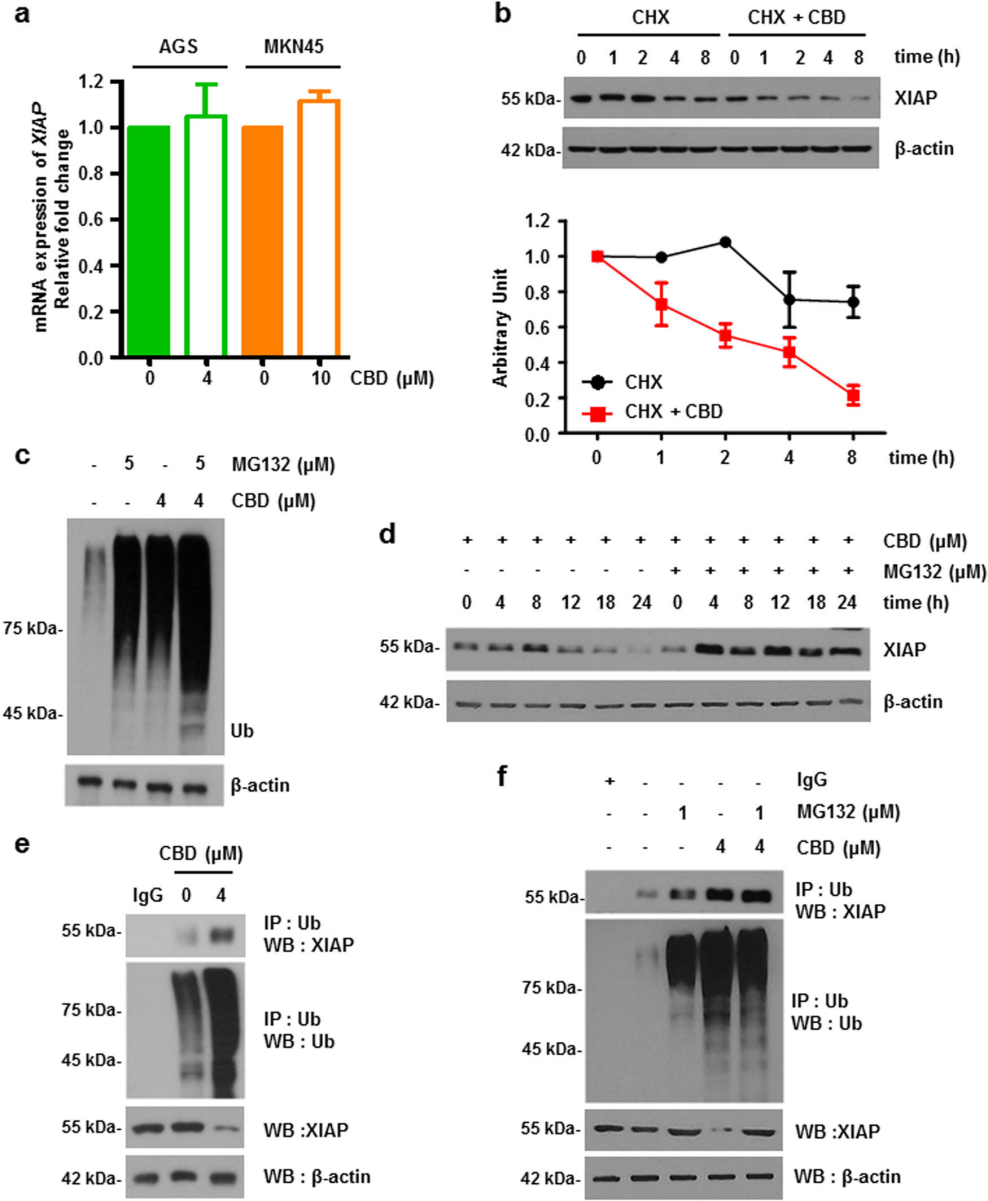
CBD reduces XIAP expression by increasing ubiquitination of XIAP via UPS. **a** Cells were treated with CBD for 24 h. Total mRNA was extracted from cells and mRNA levels of *XIAP* were assessed by qRT-PCR. **b** Effect of CBD on XIAP protein stability was determined by performing cycloheximide chase assay. AGS cells were treated with 4 μM CBD and 50 μg/ml cycloheximide at indicated times. Cell lysate was analyzed by western blot analysis. **c** AGS cells were pre-treated with 5 μM of MG132 then treated with 4 μM of CBD for 24 h. Ubiquitination was detected by immunoblotting. β-Actin was used as a loading control**. d** 4 μM CBD was treated for various time periods with or without MG132 and analyzed by western blotting. **e** AGS cells were treated with 4 μM of CBD for 24 h. Cell lysates were immunoprecipitated with IgG or anti-Ub antibodies and immunoblotted for XIAP**. f** AGS cells were non-treated or pre-treated with 1 μM of MG132 for 1 h and then treated with 4 μM CBD for 24 h. Cell lysates were immunoprecipitated with IgG or anti-Ub antibodies and immunoblotted for XIAP. **e** The interaction between XIAP and Smac was assessed by Co-IP analysis

Next, the mRNA levels of *XIAP* were measured by quantitative real-time PCR (qRT-PCR) to determine whether reduction of *XIAP* expression was modulated at the mRNA level (Fig. 3a). However, mRNA expression of *XIAP* did not change in AGS and MKN45 cell lines, suggesting that XIAP is regulated by posttranslational modification rather than at the transcriptional level. CBD attenuated the protein stability of XIAP when AGS cells were exposed or not exposed to CBD for indicated time periods during the presence of cycloheximide (CHX). As shown in Fig. 3b, CHX chase assay revealed that XIAP protein half-life was 10.43 h when AGS cells were incubated with CHX, whereas half-life of XIAP protein was 3.38 h in the CHX and CBD-treated cells, suggesting that CBD reduces the level of XIAP through ubiquitin-proteasome system (UPS). To verify this, ubiquitination was investigated via treatment of a proteasome inhibitor, MG132 (Fig. 3c). Ubiquitination increased after CBD treatment in AGS cells and ubiquitination accumulated by MG132 treatment was also further increased after CBD treatment. In addition, treatment of CBD in the presence of MG132 inhibited the reduction of CBD-induced XIAP (Fig. 3d). To determine whether the decrease in XIAP expression is an effect of increased proteasomal degradation, co-immunoprecipitation (Co-IP) was conducted in the CBD-treated condition. CBD promoted XIAP ubiquitination and MG132 significantly blocked CBD-induced XIAP degradation (Fig. 3e, f).

### SMAC activated by endoplasmic reticular stress regulates the ubiquitination of XIAP, which is associated with CBD-induced apoptosis

**Fig. 4:**
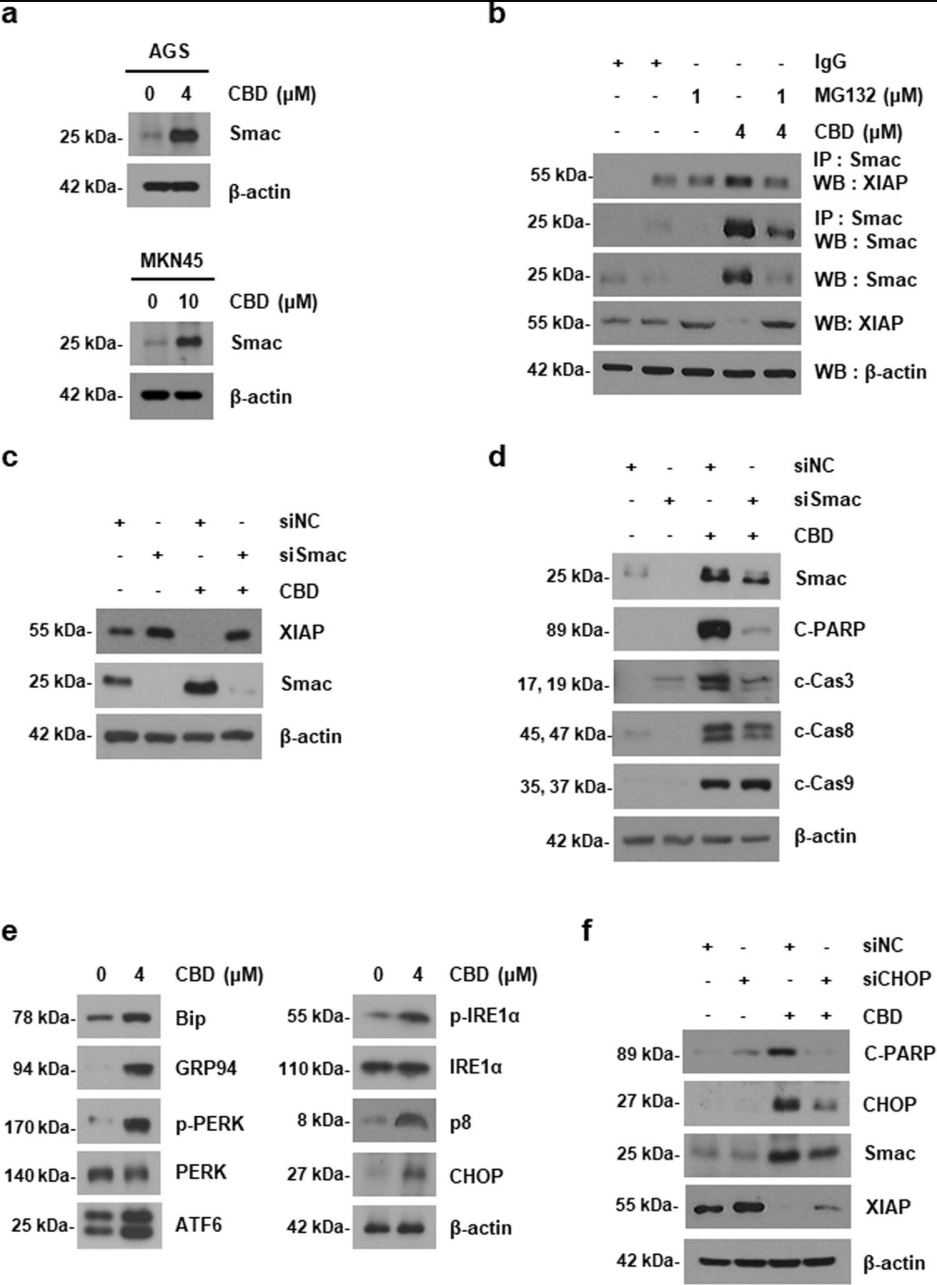
CBD enhanced Smac activation caused by ER stress and subsequent the interaction between Smac and XIAP. **a** Cells were treated with 4 μM (AGS cells) or 10 μM (MKN45 cells) of CBD for 24 h. Smac expression level was detected by western blotting analysis. **b** AGS cells were non-treated or pre-treated with 1 μM of MG132 for 1 h and then treated with 4 μM CBD for 24 h. The interaction between XIAP and Smac was determined by Co-IP analysis. **c**, **d** AGS cells were transfected with control siRNA (siNC) or Smac siRNA (siSmac). Transfected cells were treated with 4 μM of CBD for 24 h. The protein levels of Smac, XIAP (**c**), and apoptosis-related proteins (**d**) were detected by western blotting. **e** AGS cells were exposed to CBD for 24 h and then ER stress-related proteins were measured by western blotting. **f** The cells were transfected with siNC or CHOP-specific siRNA (siCHOP). The transfected cells were treated with 4 μM of CBD for 24 h. Cell lysates were immunoblotted with Smac antibody

Smac is known as a negative regulator of XIAP, a known anti-apoptotic protein^15^. We hypothesized that Smac might act on the reduction of XIAP expression. To investigate the expression of Smac, protein levels of Smac were assessed by immunoblotting in AGS and MKN45 cells (Fig. 4a). Interestingly, the protein expression level of Smac was significantly increased in both cell lines. To reveal the relationship between XIAP and Smac, we first confirmed the interaction between XIAP and Smac. As shown in Fig. 4b, binding of two proteins was elevated by CBD and MG132 decreased the interaction. Next, AGS cells were transfected with Smac siRNA. Smac knockdown partially restored CBD-induced XIAP reduction (Fig. 4c). To determine whether Smac affects apoptosis of gastric cancer cells, western blotting analysis was conducted using siRNA. Smac downregulation recovered the apoptosis induced by CBD (Fig. 4d). As Smac is reported to be regulated by endoplasmic reticular (ER) stress, we examined whether CBD induced overproduction of ER stress signals. CBD elevated ER stress-related proteins and ER chaperone proteins (Fig. 4e). Moreover, the CBD-induced Smac increase was attenuated by C/EBP homologous protein (CHOP), a transcription factor or ER stress, knockdown, whereas XIAP was increased (Fig. 4f), indicating that CBD downregulates XIAP by activating Smac through ER stress overproduction and this downregulation is caused by promoting the binding of XIAP and Smac.

### CBD leads to mitochondrial dysfunction and inducing Smac release

**Fig. 5:**
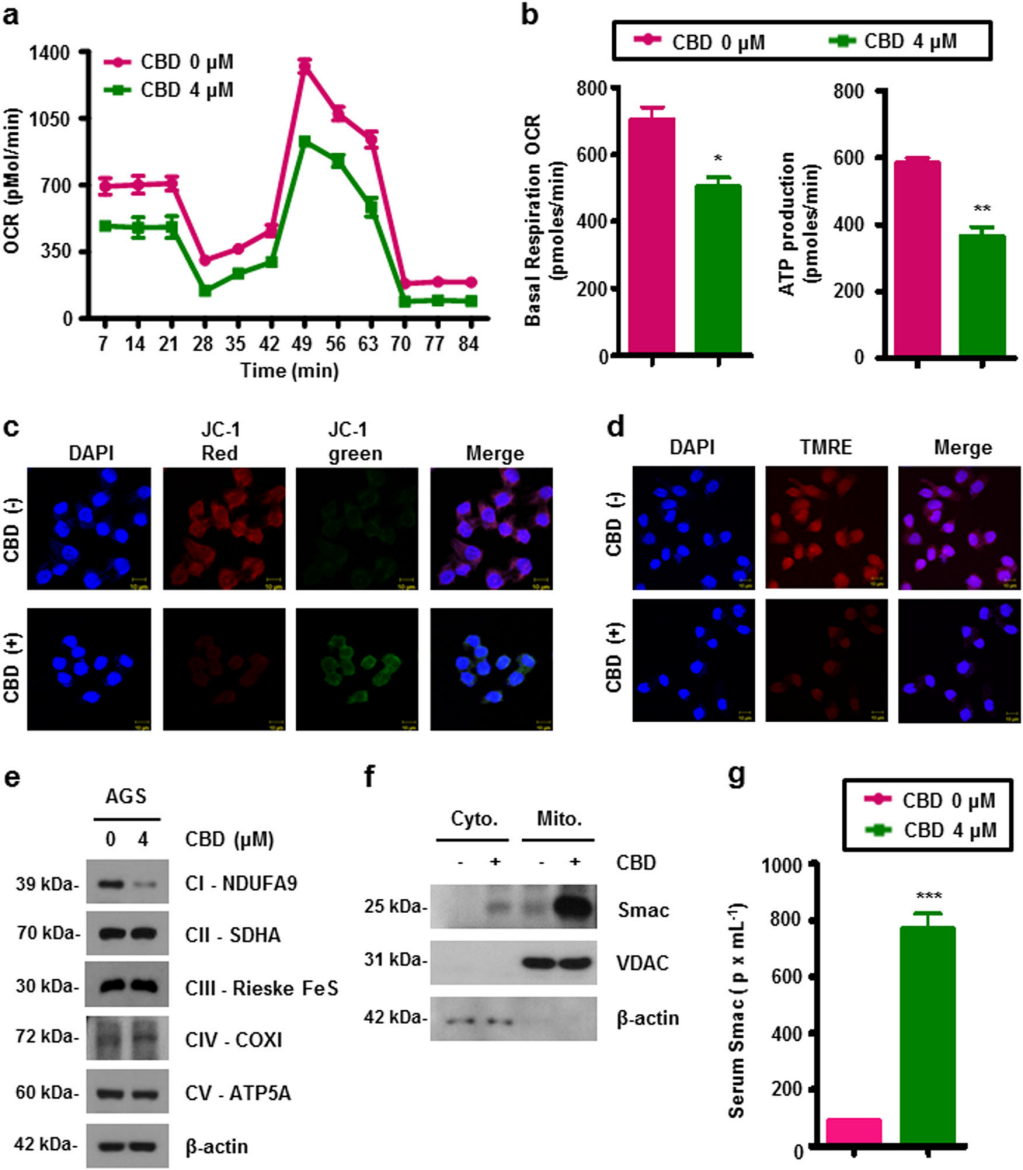
CBD promotes mitochondrial dysfunction of gastric cancer cells. **a**, **b** OCR in CBD-treated or -untreated cells was measured using an XF24 extracellular flux analyzer by adding oligomycin, CCCP, and rotenone (**a**). Basal respiration (left) and ATP production (right) were quantified using the obtained OCR (**b**). *P < 0.05 and **P < 0.01. **c** CBD-treated or untreated cells were incubated with JC-1 at 37 °C for 30 min. MMP of stained cells was determined by immunofluorescence analysis. **d** Cells were treated with 4 μM of CBD for 24 h. Treated cells were stained with TMRE dye at 37 °C for 30 min. The representative images were captured using a confocal microscope. **e** Western blotting analysis of mitochondria electron transport proteins in CBD treatment. **f** Mitochondria was isolated from CBD-treated or untreated AGS cells. Smac expression in the cytosolic and mitochondrial fractions was analyzed by western blotting. **g** Smac release was measured by using a Smac ELISA kit. ***P < 0.001

As Smac is a mitochondrial protein and its expression can be regulated by CBD, we measured oxygen consumption rate (OCR) to determine whether Smac affects mitochondrial function. The level of basal OCR was decreased in CBD-treated AGS cells (Fig. 5a). In addition, basal respiration OCR and ATP production levels were also decreased in the CBD-treated condition (Fig. 5b). Mitochondrial membrane potential (MMP) plays a key role in elimination of dysfunctional mitochondria^19^. Therefore, we then investigated the effects of CBD on MMP by staining with JC-1 dye. As shown in Fig. 5c, CBD attenuated red fluorescence and increased green fluorescence in AGS cells, indicating that MMP is inhibited by CBD. Moreover, we performed tetramethylrhodamine, ethyl ester, perchlorate (TMRE) staining to detect active mitochondria. As a result, the red fluorescence signal was reduced by CBD (Fig. 5d). Furthermore, expression of mitochondrial electron transfer proteins was assessed by western blotting after CBD treatment. As shown in Fig. 5e, expression of NADH dehydrogenase ubiquinone 1α subcomplex subunit 9 (NDUFA9), a protein associated with mitochondria complex I, was significantly reduced. Moreover, CBD increased Smac secretion to the cytosol. Interestingly, CBD also increased Smac within the mitochondria (Fig. 5f, g). Taken together, CBD affects mitochondrial dysfunction and Smac releasing in gastric cancer cells.

### CBD inhibits tumor growth in vivo mouse model

**Fig. 6:**
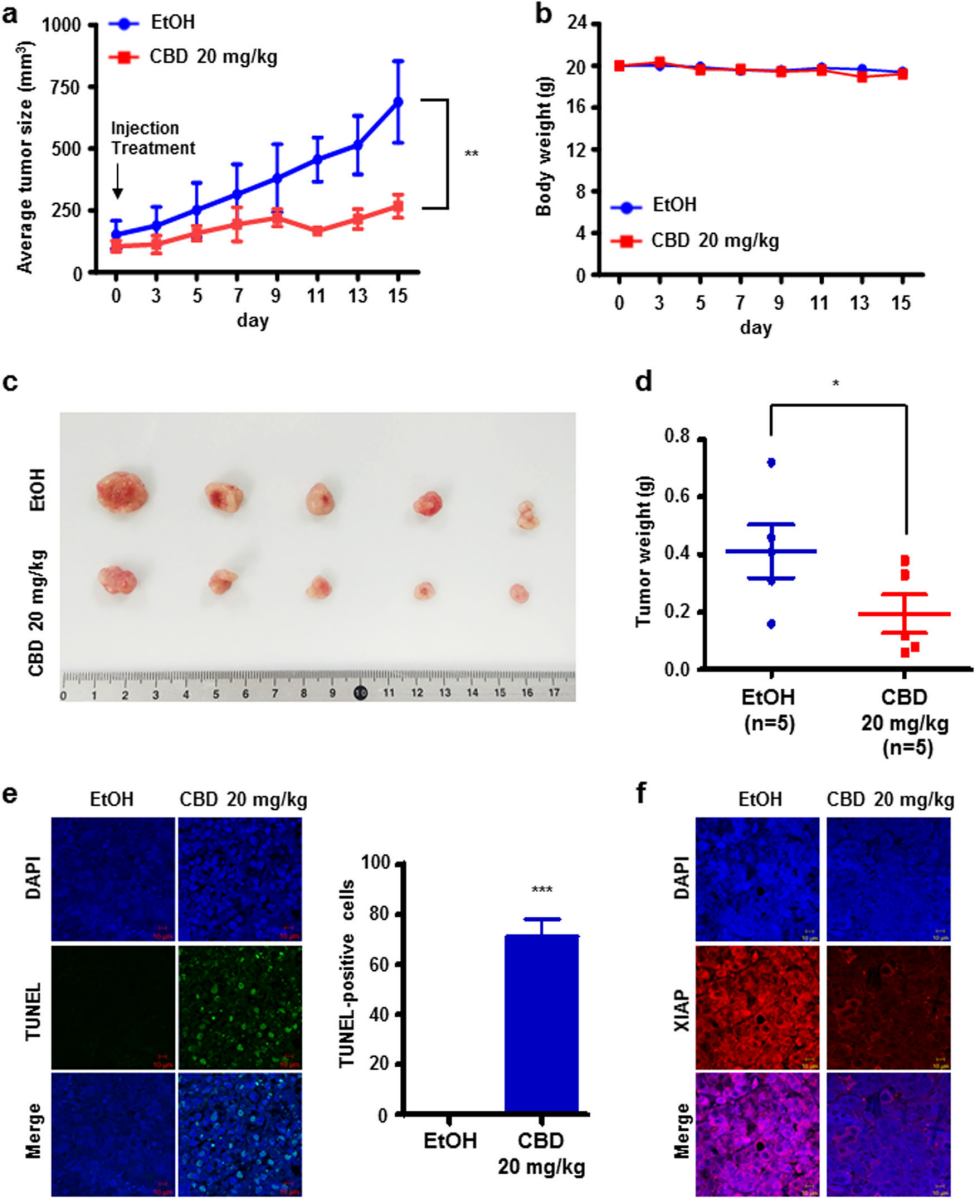
Effect of CBD treatment in an in vivo xenograft mouse model. **a** Tumor growth after subcutaneous injection in an established in vivo tumor xenograft mouse model. **P < 0.01. **b** Body weight of mice in the EtOH- and CBD-treated groups. **c**, **d** Representative images (**c**) and tumor weight (**d**) of tumor tissues from mice in the control and CBD treatment group. **P < 0.01. **e** Tumor sections were stained with TUNEL dye using In Situ TUNEL detection kit. ***P < 0.001. **f** Tumor tissue sections were immunostained with an anti-XIAP antibody (Red)

We initially assessed the antitumor effect of CBD in vitro. To investigate its antitumor effect in vivo, 4-week-old Balb/c-nude mice were subcutaneously injected with MKN45 gastric cancer cells to establish a mouse gastric cancer model. After tumor size reached 100 mm^3^, mice were injected intraperitoneally with CBD three times over the course of 1 week. The effect on tumor growth was monitored for about 15 days. As shown in Fig. 6a, tumor growth of the CBD-treated group was remarkably slower than that of the control group. After the monitoring period, tumor tissue was extracted from the mice and the size of the tumor was measured. As shown in Fig. 6c, it was confirmed that tumor size was significantly smaller in the CBD-treated group than in the control group. In addition, the mean tumor weight of the CBD-treated group was significantly less than that of the control group (Fig. 6d). However, there was hardly any difference in body weight between the control group and the CBD-treated group (Fig. 6b). A TUNEL assay was performed using tumor tissue obtained from mice to investigate whether CBD promotes apoptosis (Fig. 6e). TUNEL-positive cells (green fluorescence) were significantly increased in the CBD-treated mouse group. Moreover, expression of XIAP was decreased in the CBD-treated group (Fig. 6f). These data demonstrated that CBD suppressed tumor growth in vivo.

## Discussion

Recently, several studies on the treatment of diseases using natural products, which are safer and less toxic than existing drugs, are actively being carried out^20^. CBD, one of the major components of *Cannabis*, is becoming more popular as a treatment drug in applications such as cancer therapy, as it becomes legal to use for therapeutic purposes in many countries^21^. A number of earlier studies have suggested that the anticancer effect of CBD is associated with its ability to induce apoptotic cell death and several related signaling pathways have been reported^2,3,5^. In this study, we reported that CBD also induced apoptosis by regulating Smac/XIAP through mitochondrial dysfunction in gastric cancer for the first time.

We showed that CBD induced suppression of viability and apoptosis via caspase cascade in a dose-dependent manner in gastric cancer cell lines including AGS, MKN45, SNU638, and NCI-N87 cells, but not in the gastric normal epithelial HFE-145 cell line. In addition, CBD inhibited tumor growth and increased apoptosis in in vivo mouse models. Administration of 100 mg/kg CBD daily for 5 weeks had no side effects in the xenograft model using pancreatic cells^22^. Therefore, the 20 mg/kg dose used in the experiment is far below the dose that causes mice to have serious side effects.

One important finding of this study is that XIAP downregulation is essential for CBD-induced apoptosis in gastric cancer cells. XIAP is an important target for treatment because of its increased expression in gastric cancer patients^22^. XIAP was reduced by CBD in a dose- and time-dependent manner and overexpression of XIAP partially restored CBD-induced apoptosis. Conversely, treatment with CBD after XIAP knockdown increased apoptosis. However, flow cytometry data showed more necrosis than apoptosis. This is presumably due to excessive apoptosis leading to necrosis. Moreover, CBD significantly increased ubiquitination of XIAP, which is regulated by Smac. Smac resides in a homodimeric form in the mitochondria, where the N-terminal is truncated by stimulation such as radiation or chemotherapy and the molecule migrates to the cytosol in the form of mature Smac. Interestingly, in our system, Smac showed increased mRNA and protein levels in the mitochondria and the cytosol upon CBD treatment, implying that CBD may promote cytosolic release of Smac as well as transcription of Smac. In addition, Smac released into the cytosol by CBD treatment showed increased binding with XIAP. Thus, these findings together suggest that Smac, when promoted by CBD, translocates to the cytosol and binds to XIAP, to increase its ubiquitination, which further increases apoptotic cell death.

The ER is an important organelle for protein synthesis and modification, lipid biosynthesis, and maintenance of intracellular Ca^2+^ homeostasis^23^. When homeostasis is broken by changes in glycosylation, Ca^2+^ depletion, oxidative stress, or accumulation of misfolded or unfolded proteins in the ER lumen, and ER stress is induced^24^. During accumulation of misfolded or unfolded proteins, the three receptors in the ER transmembrane, inositol requiring enzyme-1α (IRE1α), phospho-PKR-like ER-resistant kinase (PERK), and activating transcription factor 6 (ATF6), are removed from Bip to increase the expression of ER chaperones such as Bip and Glucose Regulated Protein 94 (GRP94), then, maintained their homeostasis by eliminating them^24,25^. Moreover, in the previous study, we found that expression of ER stress-related genes is increased in CBD-treated colorectal cancer cells^5^. Consistent with this, we showed that CBD increased ER stress-related genes in gastric cancer cells. CHOP, a key transcription factor for ER stress, regulates Smac^26^. Our results showed that CBD elevated CHOP levels and knockdown CHOP using siRNA partially increased CBD-induced XIAP reduction, indicating that CBD inhibits XIAP by stimulating ER stress.

XIAP induces mitochondrial damage by regulating the Bcl-2 family proteins^27^. CBD regulates mitochondrial complex I and IV in hippocampal neurons^28^. In our data, mitochondrial complex I was downregulated by CBD; however, in contrast to the results of previous studies, OCR was remarkably reduced in CBD-treated gastric cancer cells. This different regulation of OCR by CBD treatment may require further study and might be associated with the background of other types of cells, such as neurons and cancer.

**Fig. 7:**
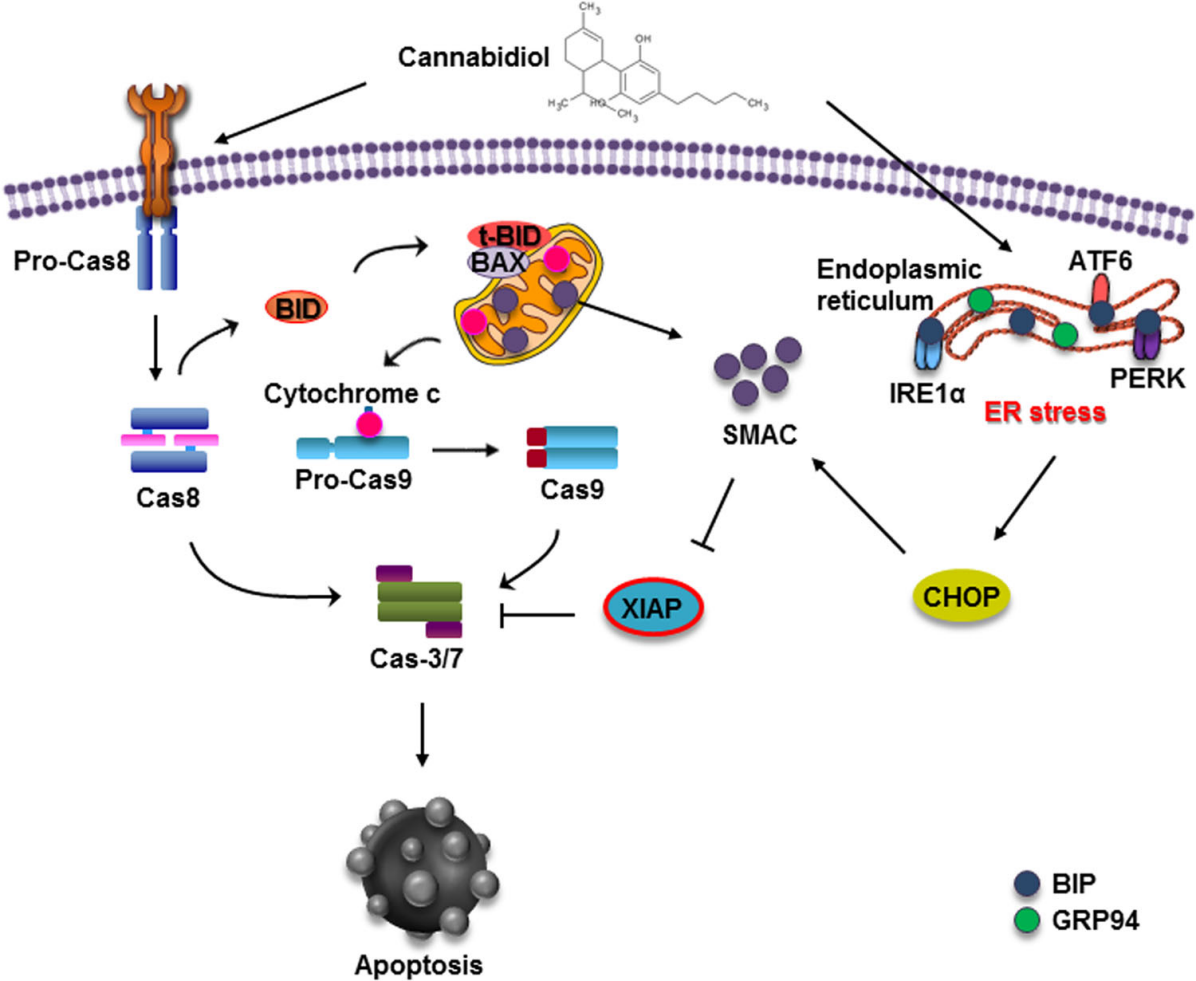
A schematic diagram of the effect of CBD treatment

In conclusion, our study showed that CBD induces apoptotic cell death in gastric cancer cells, which is triggered by ER stress generation and subsequent XIAP inhibition by Smac (Fig. 7). Taken together, our results suggest the potential of CBD in novel treatments against gastric cancer.

## Materials and methods

### Cell culture

The human gastric carcinoma AGS, MKN45, and MKN74 cells were purchased from the American Type Culture Collection (Manassas, VA, USA). Cells were maintained according to the manufacturer’s instructions. Human gastric carcinoma SNU638 and NCI-N87 cell lines were obtained from the Korean Cell Line Bank (Seoul, Korea). Gastric carcinoma cells and gastric normal HFE-145 cells were grown in RPMI1640 medium (Gibco, Grand Island, NY, USA) containing 10% fetal bovine serum (Sigma, Darmstadt, Germany) with 100 mg/mL penicillin and streptomycin (GenDEPOT, Barker, TX, USA).

### Transfection

For RNA interference, XIAP siRNA (siXIAP), Smac siRNA (siSmac), and CHOP siRNA (siCHOP) were purchased from Santa Cruz Biotechnology (Dallas, TX, USA). Cells were incubated with siRNAs and Lipofectamine RNAiMAX (Invitrogen, Carlsbad, CA) in Opti-MEM medium (Life Technologies, Darmstadt, Germany) for 6 h. After the incubation, transfection medium was exchanged with fresh cultured medium.

For overexpression of target genes, myc-XIAP plasmid and pSmac-green fluorescent protein (GFP) was obtained from Addgene. myc-XIAP plasmid and pSmac-GFP were incubated on cells with Lipofectamine 2000 (Invitrogen). After 6 h of incubation at 37 °C, expression media were replaced with fresh cultured medium.

### Reagents and antibodies

CBD was obtained from Sigma. CBD dissolved in absolute Ethanol (EtOH) was stored at −20 °C. CHX was purchased from Merck Milipore (Darmstadt, Germany). MG132 was purchased from Sigma. Antibodies against c-PARP, c-Cas3, -Cas8, and Cas9, Bid, Bax, p53 upregulated modulator of apoptosis, XIAP, Bip, GRP94, PERK, p-PERK, phospho-IRE1α, p-IRE1α, ATF6, CHOP, ubiquitin (Ub), and cytochrome c oxidase subunit I were purchased from Cell Signaling Technology (Danvers, MA, USA). Protein G PLUS-Agarose beads and antibodies against B-cell lymphoma-extra-large, NDUFA9, succinate dehydrogenase complex flavoprotein subunit A, RieskeFeS, and adenosine triphosphate synthase subunit α were purchased from Santa Cruz Biotechnology. β-Actin was purchased from Sigma. Antibody against p8 was purchased from abcam (Cambridge, UK). The secondary antibodies anti-mouse-IgG-horseradish peroxidase (HRP) and anti-rabbit-IgG-HRP were purchased from Cell Signaling Technology.

### Cell proliferation assay

Cell proliferation was determined by the WST assay using EZ-CyTox Cell Viability, Proliferation, Cytotoxicity Assay kit (DoGEN, Daeil Lab Service Co. Ltd, Seoul, South Korea). Cells were seeded at a density of 1 × 10^4^ cells per well in 96-well plates. Cells were then treated with CBD for 24 h and then treated with WST-1 for 3 h at 37 °C. Absorbance at 450 nm was measured using a microplate reader (SPECTRA190, Molecular Devices, Sunnydale, CA, USA).

### Apoptosis analysis (flow cytometry)

One of the earliest features of apoptosis is the translocation of phosphatidylserine from the inner to the outer leaflet of the plasma membrane and can be detected by binding of Annexin V^29^. Apoptosis was analyzed with an Annexin V–Fluorescein isothiocyanate (FITC) Apoptosis Detection kit (BioBud, Seoul, Korea). Cells were treated with or without CBD for 24 h and then trypsinized and centrifuged at 2000 r.p.m. for 5 min. Cells were resuspended with binding buffer. Cells were stained with 1.25 μL Annexin V–FITC reagent and 10 μL PI reagent for 30 min at room temperature (RT) in the dark. Staining was terminated and then immediately analyzed by flow cytometry (Beckman Coulter, Brea, CA, USA).

### TdT-mediated dUTP nick-end labeling assay

Cells or tissue treated with or without CBD were fixed on coverslips with 4% paraformaldehyde and permeabilized with 0.5% Triton X-100. Next, the cells were stained using an In Situ Cell Death Detection kit (Roche, Basel, Switzerland). DNA fragmentation was visualized by TUNEL assay as described by the manufacturer’s instructions. Fluorescence images were captured using a confocal microscope (Carl Zeiss, Oberkochen, Germany).

### Quantitative RT-PCR

Total RNA was isolated using TRIZOL reagent (Life Technologies). Amplification of transcripts was performed using a reverse-transcription PCR kit (Life Technologies). qRT-PCR was performed on an Applied Biosystems Quantstudio 6Flex qRT-PCR instrument using Taqman™ probes (Applied Biosystems, Foster City, CA, USA). For quantification of mRNA expression, gene expression was normalized to that of glyceraldehyde-3-phosphate dehydrogenase.

### Immunoblotting

Western blotting was carried out as previously described^30^. Immunoreactive proteins were visualized by the chemiluminescence protocol (DoGEN ECL).

### Cycloheximide chase assay

Cells were pre-treated with CBD for 24 h. Cells were collected at 0, 1, 2, 4, and 8 h after following treatment of 50 μg/mL cylcloheximide. XIAP protein stability was analyzed by western blotting analysis.

### Co-immunoprecipitation

Cells were washed with ice-cold phosphate-buffered saline (PBS) and were incubated with 300 μL lysis buffer (1 mM phenylmethylsulfonyl fluoride, protease inhibitor, and phosphatase inhibitor; Cell signaling Technology). Cells were collected and cell debris was removed by centrifugation at 15,000 r.p.m. for 5 min at 4 °C. Protein quantification was measured by performing a bicinchoninic acid assay (Thermo Scientific, Waltham, MA, USA). Supernatants were incubated with primary antibodies at 4 °C overnight. Protein G PLUS-Agarose beads were added for 1 h at 4 °C. Immunoprecipitates were washed and separated by centrifugation at 15,000 r.p.m. and heated with 2X sample buffer. Supernatants were then assessed by western blotting.

### Oxygen consumption rate

AGS cells were seeded at a density of 3 × 10^4^ cells per well in XF24 cell culture microplates (V7-PS; Seahorse Bioscience, North Billerica, MA, USA) and treated with 4 μM CBD. Cell culture medium was replaced with XF24 medium containing glucose 1 h before measurement. OCR was measured using an XF24 extracellular flux analyzer. The obtained OCR was validated by adding oligomycin (2 μg/ml), carbonyl cyanide m-chlorophenyl hydrazine (CCCP, 5 μM), and rotenone (2 μM) sequentially.

### MMP assay

MMP was assessed by staining with JC-1 (Thermo Fisher Scientific), a cationic carbocyanine dye that accumulates in the mitochondria, and TMRE (Thermo Fisher Scientific). Cells were seeded and stained with JC-1 or TMRE at 37 °C for 30 min. Images were obtained using a confocal microscope.

### Enzyme-linked immunosorbent assay

Cells were seeded and then treated with 4 μM of CBD. After 24 h incubation, cell supernatants were collected and assessed for Smac releasing a Human SMAC ELISA kit according to the manufacturer’s instruction. Human Smac ELISA kit was purchased from RayBioTech (GA, USA).

### Xenograft model

Animal experiments were performed in accordance with the guidelines approved by the Korea University Institutional Animal Care and Use Committee (KOREA-2018-0081). MKN45 cells (1 × 10^7^ cells in 100 μL of PBS) were subcutaneously injected into 4-week-old female BALB/c nude mice. Tumor size and body weight were measured three times a week and CBD was injected for the same time. When tumor size reached 100 mm^3^, five mice per group were randomly divided. The tumor volume was calculated as 0.5 × length × (width)^2^.

### Immunofluorescence

Cells were incubated at 37 °C overnight. Cells were fixed in 3.7% formaldehyde for 15 min at RT washed three times with PBS and incubated with 0.5% Triton X-100 for 15 min at RT. Cells were then incubated in blocking buffer (3% bovine serum albumin with PBS) for 1 h at 4 °C, followed by incubation with primary antibodies at 4 °C overnight. Cells were washed three times for 5 min each, after which Alexa Fluor 488-conjugated goat anti-mouse secondary antibody (Invitrogen, diluted 1:200 in PBS) or Alexa Fluor 594-conjugated goat anti-rabbit secondary antibody (Invitrogen, diluted 1:200 in PBS) was added for 17 min at 4 °C. After three washes with Tris-buffered saline containing Tween 20, cells were mounted and analyzed by confocal microscopy.

### Statistical analysis

Each assay was performed in triplicate and independently repeated at least three times. Statistical analysis was carried out using GraphPad InStat 6 Software (La Jolla, CA, USA). Statistical significance was defined as *P*-value < 0.05 (*P < 0.05, **P < 0.01, and ***P < 0.001, respectively).
